# Bilateral spontaneous non-traumatic rupture of the Achilles tendon: a case report

**DOI:** 10.1186/1752-1947-5-263

**Published:** 2011-06-30

**Authors:** Zubair Khanzada, Ulfin Rethnam, David Widdowson, Ahmed Mirza

**Affiliations:** 1 Department of Orthopaedics, Glan Clwyd Hospital, Bodelwyddan, Rhyl, UK; 2 Department of Radiology, Glan Clwyd Hospital, Bodelwyddan, Rhyl, UK

## Abstract

**Introduction:**

We present an interesting case of spontaneous non-traumatic bilateral rupture of the Achilles tendons, which is a rare condition. Delayed or missed diagnosis of Achilles tendon ruptures by primary treating physicians is relatively common.

**Case report:**

A 78-year-old Caucasian woman presented with spontaneous non-traumatic bilateral rupture of the Achilles tendons. Her symptoms started two days after she took ciprofloxacin 500 mg twice daily for a urinary tract infection and prednisolone 30 mg once daily for chronic obstructive airway disease.

**Conclusion:**

This case report aims to increase the awareness of this rare condition, which should be borne in mind with regard to patients who are on steroid therapy and are concurrently started on fluoroquinolones.

## Introduction

Spontaneous non-traumatic rupture is rare and is commonly associated with long-term use of corticosteroids [1] or fluoroquinolones [2]. When prescribed together, steroids and fluoroquinolones can have a potentiating effect, causing an increase in the risk of Achilles tendon rupture [3]. Bilateral spontaneous Achilles tendon rupture is extremely rare, with fewer than 20 cases reported in the literature [4]. We present an interesting case of spontaneous bilateral Achilles tendon rupture.

## Case report

A 78-year-old Caucasian woman presented to the Accident and Emergency Department with spontaneous onset of severe pain in both ankles. There was no history of trauma. The patient was given oral ciprofloxacin hydrochloride 500 mg twice daily for urinary tract infection. She was also given oral prednisolone 30 mg once daily for chronic obstructive airway disease. Two days after starting the medications the patient developed intense bilateral ankle pain. She was unable to walk. The symptoms started on the left side first, followed by the right side a few hours later. There was nothing in the patient's history to suggest chronic Achilles tendinopathy.

At the initial assessment, the patient was unable to bear weight because of pain. Both ankles appeared to be swollen with bruising over the Achilles tendon region. There was tenderness over both Achilles tendons near their insertions into the calcaneus with palpable gaps in the substance of the tendons. She had a positive Thompson's test and was unable to perform active plantar flexion with either ankle joint. There was no neurological deficit distally.

**Figure 1:**
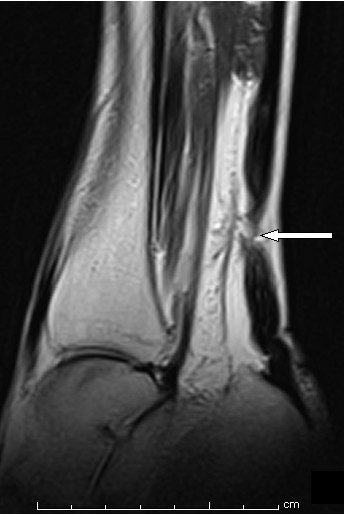
**Sagittal view magnetic resonance imaging (MRI) scans of the patient's right ankle showing rupture of the Achilles tendon**.

**Figure 2:**
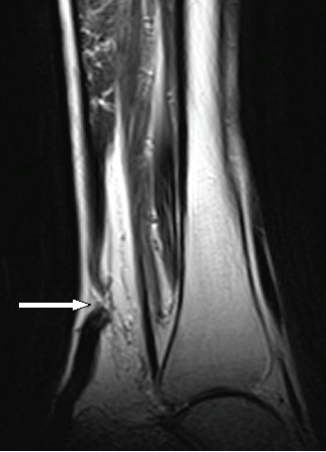
**Sagittal view MRI scans of the patient's left ankle showing rupture of the Achilles tendon**.

A clinical diagnosis of bilateral spontaneous rupture of Achilles tendon was suspected. Because of the rarity of the suspected diagnosis, a differential diagnosis of deep vein thrombosis (DVT) was also taken into consideration. A Doppler imaging study was obtained to rule out DVT, which proved to be negative. Magnetic resonance imaging (MRI) scans were obtained for both ankles, which confirmed bilateral Achilles tendon rupture 5 cm proximal to insertion into the calcaneus (Figures 1 and 2). There were no features suggestive of pre-existing tendinopathy on the MRI scans.

A decision to apply conservative management was made in consideration of the patient's age, co-morbidities and activity level, as well as the patient's wishes. Steroids and fluoroquinolones were stopped as they were believed to be the causative factor and can interfere with the tendon-healing process.

The patient was placed in bilateral below-knee plasters in gravity equinus for four weeks, in mid-equinus for two weeks and in a neutral position for two weeks. The patient was followed up at four, eight and 12 weeks. At 12 weeks, both the Achilles tendons had healed. On palpation, the tendons were in continuity, with no gap at the area of the rupture. An assessment of ankle range of movement revealed dorsiflexion of 40° and plantarflexion 30° bilaterally. The patient's American Orthopaedic Foot and Ankle Score *(*AOFAS) for foot and ankle disorders for her hind foot had improved from 18 on presentation to 61 at the final follow-up examination. The patient was able to bear weight and mobilize with a stick. Some stiffness in both ankle joints continued as residual symptoms, for which physiotherapy was continued.

## Discussion

The Achilles tendon is the tendinous extension of three muscles in the lower leg: the gastrocnemius, the soleus and the plantaris. It is the thickest and strongest tendon in the body. It is inserted into the middle part of the posterior surface of the calcaneum. The primary function of the Achilles tendon is to transmit the power of the calf to the foot, enabling walking and running. Achilles tendon ruptures account for 20% of all large tendon ruptures [4].

Achilles tendon tears are usually traumatic, resulting from a large force on a normal tendon or a physiological force on a weak tendon. The mechanism usually involves eccentric loading on a dorsiflexed ankle with the knee extended (soleus and gastrocnemius on maximal stretch). The majority of tears occur in the watershed area, an area of structural weakness located approximately 6 cm proximal to the tendon insertion on the calcaneus [5].

In most cases reported in the literature, bilateral spontaneous rupture of the Achilles tendon has been associated with corticosteroid use. The exact mechanism by which corticosteroids cause tendon damage is not clear. It is said that steroids have the ability to alter the collagen structure of tendons by contributing to dysplasia of collagen fibrils, thus reducing the tensile strength of the tendon [6]. Corticosteroids can also interfere with collagen fiber cross-linking, which can lead to disruption in the normal healing process of the tendon [1,6,7].

The other association of spontaneous rupture of the Achilles tendon is with the use of fluoroquinolones [8,9]. Van der Linden *et al*. [10] described bilateral Achilles tendon ruptures two, three and six days after initial treatment with fluoroquinolones and bilateral Achilles tendinitis one, two, and three days after initial treatment with fluoroquinolones. Animal studies have suggested that chelation of magnesium and free radical formation result in oxidative stress, leading to a direct toxic effect on collagen [11-14].

The reported incidence of spontaneous Achilles tendon rupture is 0.02% in the Western population. Less than 1% of patients have simultaneous bilateral rupture [4].

Our case report is of interest because the patient had only a short course (two days) of fluoroquinolones and oral steroids. Her initial presentation did take us by surprise. With conservative treatment, the final outcome was good. This rare condition can be easily missed if one is not aware of the possibility of spontaneous rupture of the Achilles tendon with the concurrent use of steroids and fluoroquinolones.

## Conclusion

This case report aims to increase the awareness of the risk of this rare condition in patients who are started on steroids and fluoroquinolones concurrently even for a short period.

## Consent

Written informed consent was obtained from the patient for publication of this case report and any accompanying images. A copy of the written consent is available for review by the editor-in-chief of this journal.

## Competing interests

The authors declare that they have no competing interests.

## Authors' contributions

ZK made substantial contributions by identifying, writing and carrying out the literature search. UR was involved in critically revising the case report. DW helped in performing the imaging and made the imaging studies. AM gave final approval of the manuscript version to be published.

All authors have read and approved the final manuscript.
